# Substrate stiffness effect on molecular crosstalk of epithelial-mesenchymal transition mediators of human glioblastoma cells

**DOI:** 10.3389/fonc.2022.983507

**Published:** 2022-08-25

**Authors:** Bernadette Basilico, Ilaria Elena Palamà, Stefania D’Amone, Clotilde Lauro, Maria Rosito, Maddalena Grieco, Patrizia Ratano, Federica Cordella, Caterina Sanchini, Silvia Di Angelantonio, Davide Ragozzino, Mariafrancesca Cascione, Giuseppe Gigli, Barbara Cortese

**Affiliations:** ^1^ Institute of Science and Technology Austria (ISTA), Klosterneuburg, Austria; ^2^ National Research Council-Nanotechnology Institute (CNR Nanotec), Lecce, Italy; ^3^ Department of Physiology and Pharmacology, Sapienza University, Rome, Italy; ^4^ Center for Life Nanoscience, Italian Institute of Technology (IIT), Rome, Italy; ^5^ National Research Council-Nanotechnology Institute (CNR Nanotec), Rome, Italy; ^6^ Department of Mathematics and Physics “Ennio De Giorgi” University of Salento, Lecce, Italy

**Keywords:** Mechanotaxis, cellular microenvironment, glioblastoma, molecular pathways, stiffness

## Abstract

The complexity of the microenvironment effects on cell response, show accumulating evidence that glioblastoma (GBM) migration and invasiveness are influenced by the mechanical rigidity of their surroundings. The epithelial–mesenchymal transition (EMT) is a well-recognized driving force of the invasive behavior of cancer. However, the primary mechanisms of EMT initiation and progression remain unclear. We have previously showed that certain substrate stiffness can selectively stimulate human GBM U251-MG and GL15 glioblastoma cell lines motility. The present study unifies several known EMT mediators to uncover the reason of the regulation and response to these stiffnesses. Our results revealed that changing the rigidity of the mechanical environment tuned the response of both cell lines through change in morphological features, epithelial-mesenchymal markers (E-, N-Cadherin), EGFR and ROS expressions in an interrelated manner. Specifically, a stiffer microenvironment induced a mesenchymal cell shape, a more fragmented morphology, higher intracellular cytosolic ROS expression and lower mitochondrial ROS. Finally, we observed that cells more motile showed a more depolarized mitochondrial membrane potential. Unravelling the process that regulates GBM cells’ infiltrative behavior could provide new opportunities for identification of new targets and less invasive approaches for treatment.

## Introduction

While relatively rare, glioblastoma (GBM) is one of the deadliest of human cancers with a median survival of 12-15 months, mostly caused by relapse (1). Recurrence in GBM patients is, unfortunately, inevitable. Even with maximal resection, GBM can be traced in adjacent and high distant sites in the brain (2). In fact, GBM cells typically do not to metastasize in the classical way but infiltrate into the surrounding tissue and brain parenchyma through degradation of the extracellular matrix (ECM) or squeezing through brain interstitial spaces (3). Recently the perception that GBM tumors are associated with severe alterations of the stiffness of the surrounding milieu has emerged (4–7). More and more studies report GBM cells to move more rapidly on stiffer substrates, failing to migrate effectively on substrates with elastic moduli akin to the brain parenchyma (4, 7). Thus, while it is evident that mechanical signaling from the ECM is a key regulator of GBM invasion, this called into questioning how intracellular signaling, and the associated biochemical cascade, can selectively contribute to cell migration under different mechanical cues.

The invasive migratory behavior of cells commonly occurs within the framework of the epithelial-mesenchymal transition (EMT) (8). As the tumor develops and progresses, epithelial cells undergo a cadherin switching, losing their characteristic polarity whilst interchanging among different cadherin isoforms at the cell–cell junctions. Enhanced invasive behavior of cells has therefore been associated with the activation of signaling pathways (such as TGF-β or Wnt/β-catenin) which in turn triggers downregulation of the epithelial cell surface markers and cytoskeleton components (such as E‐cadherin) while promoting expression of mesenchymal markers (i.e., fibronectin, vimentin and N-cadherin) (9, 10). However, contradictory results were also reported, showing that downregulation of N-Cadherin is correlated with a faster and less persistent migration of normal neural and GBM cells (11, 12). These discrepancies have been associated with differences between N-Cadherin, mRNA and protein expression levels (13).

Cadherins and Catenins-related pathways have also been identified as mechanosensitive (14, 15). Previous studies reported that decreasing stiffness of the ECM leads to increase in E-cadherin and N-cadherin expression with consequent loss of cell–cell adhesion in hepatocytes and pancreatic cancer cells (16–18). Conversely, human embryonic stem cells displayed very low levels of E-cadherin on soft microposts (19). As Catenins play a key role in the cadherin-mediated cell adhesion, activation of Wnt/β-catenin signaling in GBM cells has been also reported in correlation to increased substrate stiffness (20).

A synergistic interdependence between substrate stiffness and the epidermal growth factor receptor (EGFR) has also been described, showing that increasing stiffness can regulate morphology and migration of GBM cells (7, 21, 22). An increasing number of findings also suggest a crosstalk interplay among reactive oxide species (ROS) and cadherins, such as E- and N-cadherin, and EGFR (22, 23). Most studies have also implied a complex interdependence between dynamic changes in mitochondrial morphology and ROS generation with the change of the rigidity of the microenvironment (24–26). For instance, GBM cells on substrates of increasing stiffness reported an alteration in mitochondrial activity in function of the mechanical properties of the substrate (27). In particular, higher stiffnesses showed to decrease ROS intracellular levels (28), and to amplify endothelial ROS (29). However, correlation of all the aforementioned elements to the mechanical cues and differences in motility on comparable substrates is yet to be reported. A better understanding of the interplay between the cells’ mechanical environment and cell dynamics without varying material properties such as surface energy and chemistry could help understand cellular processes underlying cell communication dictated by cell-cell and cell-substrate interactions.

Recently, we observed that the different rigidity of the microenvironment triggered cell motility of GBM cells relative to the cell phenotype. The aim of this study is to further investigate the EMT-related changes of human derived-GBM cells, specifically U251 and GL15, and to study the integrated network of biochemical and biomechanical events, involving changes in E- and N-Cadherin, β-catenin, EGFR expression and ROS production relatively to the change of migration speed to the different stiffnesses. We then show that the decrease in the rigidity of the substrate can promote elements of EMT, including increase of N-Cadherin and EGFR expression which was not correlated to the invasiveness. Additionally, we show that substrate stiffness alters actin fiber reorganization, the mitochondrial morphology, and changes in cell shape towards a mesenchymal phenotype. Establishing what triggers these changes solely due to the mechanical properties of a substrate disregarding the chemistry, will allow us to understand how the rigidity of the microenvironment regulates the EMT molecular pathway to promote tumor invasion and elucidate functional implications of these findings.

## Materials and methods

### Substrate fabrication

Two commercially available PDMS, Sylgard 527 gel and Sylgard 184 elastomer (Dow Corning), were used to create PDMS substrates with variable mechanical properties. Sylgard 527 was selected in this work, as a soft substrate, due to its very low elastic modulus (<10 kPa) within the physiological range of elastic moduli of the *in vivo* brain tissue (30). Once mixed (5:4), the PDMS 527 was poured into 35 mm diameter petri dishes to create ∼1-2 mm thick films. For the Stiff substrates a 50:1 mixture of Sylgard 184 was spin coated onto a slab of 1mm thick of PDMS 10:1 (Sylgard 184) to create ∼10 µm thick film. PDMS substrates were cured at 65°C overnight (12–24 hours) before all experiments. Substrates were washed with 70% ethanol and DI water and sterilized with UVB before seeding.

### Young’s modulus measurements

PDMS substrates were characterized mechanically with atomic force microscope (AFM) (Bioscope Catalyst, Bruker Inc. USA), mounted on an inverted optical microscope (Zeiss Observer Z1, Zeiss GERMANY). AFM experiments were performed in force-volume (FV) mode by using the RTESPA probes (Bruker Inc. USA), having a nominal spring constant of 5 N/m. Parameters used in each FV experiment were: Scan area 10 µm, Ramp rate 3 Hz, FV scan rate 0.05 Hz, Trigger Threshold 100 nm, Number of samples 256, Sample per line 64. To estimate Young’s modulus of the substrates, the recorded force-distance curves were analyzed by Nanoscope Analysis software (Bruker Inc. USA), in accordance with Sneddon model, modified in order to take into account adhesion; statistical significance of obtained results were evaluated by a one-way ANOVA test.

### Contact angle

The contact angle of each PDMS substrate was determined *via* a static sessile drop technique using a contact angle goniometer (Dataphysics OCA 20), using a 3 μl drop of water. Each substrate was tested for contact angle after approximately 10 s of dropping water onto the surface to ensure the droplet was static, for at least six times at different points on the sample. An average of 3 independent measurements for each condition was used to determine the contact angle of each PDMS substrate.

### Protein adsorption measurements

To analyze differences in water contact angles of substrates with different protein adsorption, substrates were immersed in protein solutions of Poly-L-Lysine (PLL) and collagen type I (COL1) (Sigma-Aldrich CO, St. Louis, MO, USA). Specifically, Poly-L-lysine solution (0.01%, Sigma-Aldrich) was prepared by dilution in distilled water. COL1 (5 mg/ml), was prepared from calf skin (Sigma Aldrich) in acetic acid and diluted in distilled water. Both PLL and COL 1 were poured on the sample and incubated for 24 h at 37 °C. Finally, the protein solution was removed, and the samples were washed twice.

Protein concentration was measured using the DC Protein Assay Kit (Bio-Rad, Hercules, CA) according to manufacturer instructions. All the experiments were performed in triplicate. Following incubation, samples were analyzed using a Glomax Discovery microplate reader.

### Cell cultures

Human GBM cell lines U251-MG(ATCC) and GL15 (kindly provided by Dr. Emilia Castigli, Perugia University) were used for all experiments because of their highly invasive nature. Cells were grown in Dulbecco’s modified Eagle’s medium (DMEM, Gibco) supplemented with 10% heat-inactivated fetal bovine serum (FBS, Gibco), 100 unit/ml penicillin G sodium and 100 μg/ml streptomycin sulfate at 37°C in humidified air with 5% CO_2_. Cells were seeded at a density of 4000 cells/cm^2^ and were analyzed 24h after seeding.

### Analysis of cell morphology and migration

Cells were counterstained with rhodamine-conjugated phalloidin to visualize cytoskeletal F-actin filaments and captured using a confocal microscopy system (Olympus) equipped with a 40× (UPlanFLN, NA 1.30, oil) and 60× (UPlanSApo, NA 1.35, oil) with a resolution of 1024 × 1024 pixels. Cell morphology was characterized using the particle measurement feature within ImageJ (www.nih.gov) to obtain spread area, circularity, aspect ratio (A.R.) and Feret’s diameter of single cells. Circularity of cells was calculated as = 4π (area/perimeter^2^). Values of 1.0 designate a perfect circle, and values near zero are an indication of a more elongated morphology of cells.

Time-lapse imaging was conducted on an Olympus IX73 inverted microscope, equipped with a QImaging OptiMOS sCMOS camera (QImaging, Surrey, BC, Canada) and a stage-mounted incubator with CO_2_ and temperature control (H201; Okolab, Pozzuoli, Italy). Bright field images were acquired every 2 min using a 10× (Plan N, NA = 0.25, Ph1) or 20× (LUCPlan FLN, NA = 0.45, Ph2) objective over 8 h. Cell migration was assessed using the manual tracking plugin (mtrackj) for Fiji software. In brief, instantaneous speed was determined by finding the is the length travelled by individual cells divided by time between cell positions in each frame.

### Immunofluorescence and quantitative image analysis

Cells on the different substrates were fixed using 4% paraformaldehyde in PBS for 20 min at room temperature before being permeabilized with 0.2% (v/v) Triton X-100 in PBS for 5 minutes and blocked with PBS containing 1% BSA. Cells were then incubated with total EGFR (Cat#D38B1, 1:200, Cell Signaling, Danvers, MA), E-cadherin (Cat#610182, BD Biosciences, 1:100), phalloidin-TRITC (Sigma) (1:500) (for F-actin labelling) in blocking buffer. Fluorescent dye (DYE-Light)-conjugated secondary antibodies against goat IgG were used at a dilution of 1:500 for 1 hour at 37°C in blocking buffer. After washing in PBS the samples were mounted with HOECHST 33258 (Sigma), 1mg/ml in PBS 1X for 5min. Cells were viewed on a Confocal (Olympus) microscopy system equipped with a 40X (UPlanFLN, NA 1.30, oil) and 60X (UPlanSApo, NA 1.35, oil).

The total cell fluorescence was calculated using ImageJ software. A ROI was drawn around each individual cell and the total corrected fluorescence was calculated as: corrected total cell fluorescence (CTCF) = integrated density - (area of selected cell X mean fluorescence of background readings) and subsequently normalized.

### Western blotting analysis

For protein analysis, cells were seeded on 24 well plates (6 × 10^5^ cells); cells were washed with PBS and lysed in hot 2× Laemmli buffer, boiled 5 min and sonicated. The same amount of proteins was separated on 8.75% SDS-polyacrylamide gel electrophoresis and analyzed by western immunoblot using the following primary antibodies: E-Cadherin (1:200, Santa Cruz Biotechnology) N-Cadherin (1:2000, Millipore), EGFR (1:1000, Cell Signaling), beta-catenin (1:2000, Sigma-Aldrich), actin (1:5000, Sigma-Aldrich); HRP-tagged goat anti-mouse and anti-rabbit IgG were used as a secondary antibody (1:2000; Dako). Detection was performed through the chemiluminescent assay Immun-Star Western C Kit (Bio-Rad, CA) and densitometric analysis was carried out with Quantity One software (Bio-Rad, CA).

### Measurement of mitochondrial mass and membrane potential

Mitochondrial mass localization was obtained by first loading cells with MitoTracker Green FM MitoTracker Green FM (M7510 Thermofisher, 200 nM), for 30 min, after which they were fixed and imaged with confocal microscopy. To quantitatively assess cellular mitochondrial networks, a semi-automated ImageJ plug-in Mitochondrial Network Analysis (MiNA) toolset was used (31). Briefly, images were filtered and skeletonized for mitochondrial network. The resulting mitochondrial skeleton was vectorized to identify and count/measure mitochondrial morphology, such as mean length of branches, mean network size (the mean number of branches per network), and mitochondrial footprints (mitochondrial coverage area).

Mitochondrial membrane potential (ΔΨm) was evaluated with the potentiometric dye JC-1 (5,5′,6,6′-tetrachloro-1,1′,3,3′-tetraethylbenzimidazolo-carbocyanine iodide; Carlo Erba). Cells were seeded on the different substrates and allowed to attach for 24h. The media was then replaced with fresh media containing JC-1 (5μg/ml) and cells were incubated for an additional 30 min at 37°C in the dark. Following loading, cells were washed and imaged immediately in NES. Fluorescence intensity was monitored and on the confocal microscope (Olympus) with a 40x (UPlanFLN, NA 1.30, oil) objective. Fluorescence was excited using the 488 nm line of an Argon-ion laser. The JC-1 dual fluorescence-emission of the green and red fluorescence was collected simultaneously. The sensitivities of the separate emissions channel photomultipliers were identical to allow a relative comparison of the red and green fluorescence. Intensities of the red and green fluorescence from the images were measured using Image J after background subtraction.

### Measurement of reactive oxygen species

Cytosolic ROS levels were measured using 2′,7′-dichlorofluorescein diacetate (DCFH-DA; Sigma-Aldrich, 35845) according to the manufacturers’ instructions. Briefly, cells were washed with PBS, and then incubated for 45 min with DCFH-DA (10 μM) at 37°C.

Mitochondrial superoxide production was determined using MitoSOX red (ThermoFisher, M36008) a mitochondrial superoxide indicator. Cells were loaded with 5 μM MitoSOX red in HBSS with Ca^2+^/Mg^2+^and incubated at 37°C. Images were obtained by means of a confocal microscope (Olympus) with a 40x oil objective. Fluorescence intensity was analyzed using a microplate reader Glomax Discovery (Promega). Positive controls were performed by adding H_2_O_2_ to a final concentration of 100 μM to the substrate that contained the experimental buffer and measuring the resulting fluorescence.

### Statistical analysis

Data were analyzed using Sigma Plot (Systa Software, Inc., San Jose, CA, United States). Data are presented as mean ± standard error of the mean (SEM). Unpaired Student’s t test was used as indicated in the figure legend, and differences were considered statistically significant when p ≤ 0.05. Differences among multiple treatment groups were assessed by analysis of variance (ANOVA) followed by a Holm-Sidak *post hoc* test. Differences were considered significant with p ≤ 0.05.

## Results

### Substrate characterization

**Figure 1:**
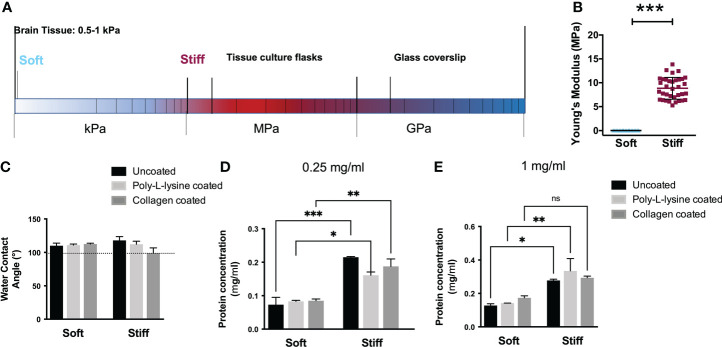
Characterization of the surface properties of the substrates ranging from soft to stiff. **(A)** Schematization of the Young’s modulus compared with the elasticity of brain tissue. **(B)** Elastic modulus of the substrates (n = 3 samples for each stiffness) in the range from few kPa to ~12 MPa. Student T test, ***p<0.001. **(C)** Water contact angle of uncoated and protein coated (poly-L-lysine and collagen I) substrates. The water contact angles of the uncoated substrates (black) are approximately 110°, indicating a similar surface energy and hydrophobicity. The water contact angles of protein coated substrates showed comparable values, indicative of similar protein adsorption behavior and surface energy. **(D-E)** Protein concentration (mg/mL) determined on uncoated and protein coated (poly-L-lysine and collagen I) Soft and Stiff substrates for different BSA protein concentration: 0,25mg/ml **(D)** 1mg/ml **(E)**. Data presented as mean ± S.D., n = 3 independent experiments for each condition. One-way ANOVA followed by Holm-Sidak post-hoc analysis; *p<0.05, **p<0.01, ***p<0.001. ns = not significant.

Tissues in the body exhibit different mechanical properties (**Figure 1A**), with a wide range of stiffnesses (32) influencing cell migration. Although the key role of a cell’s mechanical environment has been widely established, most GBM *in vitro* studies have been conducted on traditional tissue culture plastic (TCP) which presents a Young’s modulus in the range of MPa. Herein, we chose to use PDMS substrates with stiffness comparable to the TCP to serve as the baseline for comparison and to rule out the influence of the different chemistries of the substrate, comparing similar materials. Therefore we used Sylgard 184 (E = 12.6 MPa, **Figure 1B**) topped with a thin layer of PDMS (50:1) referred herein as Stiff substrates (4). PDMS substrates with stiffness similar to that of normal brain tissue and glioma tumors were obtained using Sylgard 527 (33, 34) (5:4, E ~ 1.5 kPa, **Figure 1B**), referred as our Soft substrates. This is in fact consistent with the rigidity of the environment in which GBMs infiltrate, which ranges from 0.1 to 10 kPa (35).

As the surface energy of a substrate can influence the kind and amount of protein absorption on the surface, we evaluated the wettability of the substrates to determine whether there was a difference between the PDMS formulations that might affect cell adhesion. Water contact angles (WCA) measurements of the substrates reported in **Figure 1C**, depicted the hydrophobic nature of all PDMS formulations with values ranging from ∼ 110°± 3.9 (for the Soft substrates) to 118°± 5.7 (for the Stiff) which were statistically equivalent. Similarly, coating the substrates with adhesion molecules such as collagen, or poly-L-Lysine did not show significant changes in the values of the WCA, comparable to the uncoated ones, suggesting a comparable protein adsorption. Substrates coated with poly-L-lysine presented water contact angles between 110.9°± 1.6 (Soft) and 112.2°± 4.5 (Stiff). Substrates coated with collagen I showed a contact angle of ∼113° for Soft substrates while it slightly decreased to ∼106° for the Stiff, without showing significant statistical differences.

As cells bind to extracellular matrix proteins adsorbed onto the surface, we also analyzed protein adsorption, as shown in **Figure 1D**. No significant difference in protein adsorption was observed among coated and uncoated substrates on the Stiff and on Soft formulations in accordance with the wettability data. However, a higher adsorption level was observed on stiffer respect to Soft (36). These measurements corroborate the use of substrates without protein coating to rule out the effect of proteins and to examine the cell behavior solely due to the mechanical rigidity of the substrates.

### Stiffness regulates cell spreading and modulates cell motility

**Figure 2:**
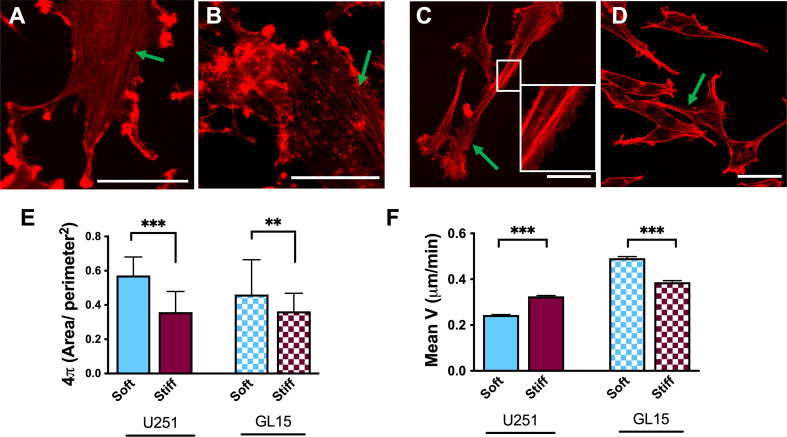
Effects of rigidity on cytoskeletal re-organization and motility in U251-MG and GL15 cells. **(A–D)** Representative confocal images of U251-MG cells stained with phalloidin-TRITC on Soft **(A)**, and Stiff **(B)** substrates and GL15 cells on Soft **(C)** and Stiff **(D)** substrates. The green arrows indicate stress fibers. The inset in (c) highlights the presence of pseudopodia along the cell body. Scale bar 50 µm. **(E)** Quantification of circularity of U251-MG and of GL15 on different substrates. Data presented as mean ± S.D. **(F)** Quantification cell motility of U251-MG and of GL15 on different substrates. Data presented as mean ± S.E.M.; n = 3 independent experiments for each condition. Student’s t-test was used for statistical analysis; **p<0.01, ***p<0.001.

To investigate whether differences in the rigidity of the substrates relate to the way cells respond and adhere to their microenvironment, we examined the cell morphology and area using phalloidin staining, to visualize filamentous actin (F-actin) of the GBM cells. The two GBM cell lines (U251-MG and GL15) plated on the above-mentioned substrates, confirmed a heterogeneity of the cells, showing unique features. Specifically, U251-MG showed a well-spread polygonal morphology on all substrates, displaying well-ordered stress fibers spanning the cytoplasm (as indicated by the green arrows, in **Figures 2A, B**), with peripheral ruffles around the edge of the cell on Soft substrates (**Figures 2A, B**) and an increase in membrane ruffles over the surface of the cell, on stiffer substrates.

GL15 cells instead, displayed a relatively elongated morphology with long extensions of stress fibers, as shown in **Figures 2C, D**. Moreover, along the sides of the body of the GL15 cells on the Soft substrates, we observed an elevated presence of lamellipodia or filopodia, as shown in the inset of **Figure 2C**, while on the Stiff substrates, the polymeric actin was organized into stress fibers (**Figure 2D**), as indicated by the green arrows. Quantification of the morphological features reported in **Figure 2E**, showed that both cell types exhibited a rounder morphology on Soft substrates respect to Stiff.

Because membrane ruffles are often observed at the leading edge of migrating cells (37), we evaluated the dynamic behavior of GBM cells through their trajectory from time-lapse acquisitions. The cell types displayed opposite behavior of speed rates on softer substrates respect to Stiff (**Figure 2F**) with U251-MG cells showing a decrease of velocity from 0,32± 0,003 µm/min on the Stiff to 0,24 ± 0,002 µm/min on Soft substrate. Whereas the motility of GL15 cells increased with the decreasing the rigidity of the substate showing a motility of 0,38± 0,007 µm/min on the Stiff to 0,49 ± 0,006 µm/min on Soft. Collectively, these data confirm that our substrates stiffness regulates cell morphology and motility and that it is phenotype dependent.

### E-Cadherin and N-Cadherin expression are influenced by substrate stiffness

**Figure 3:**
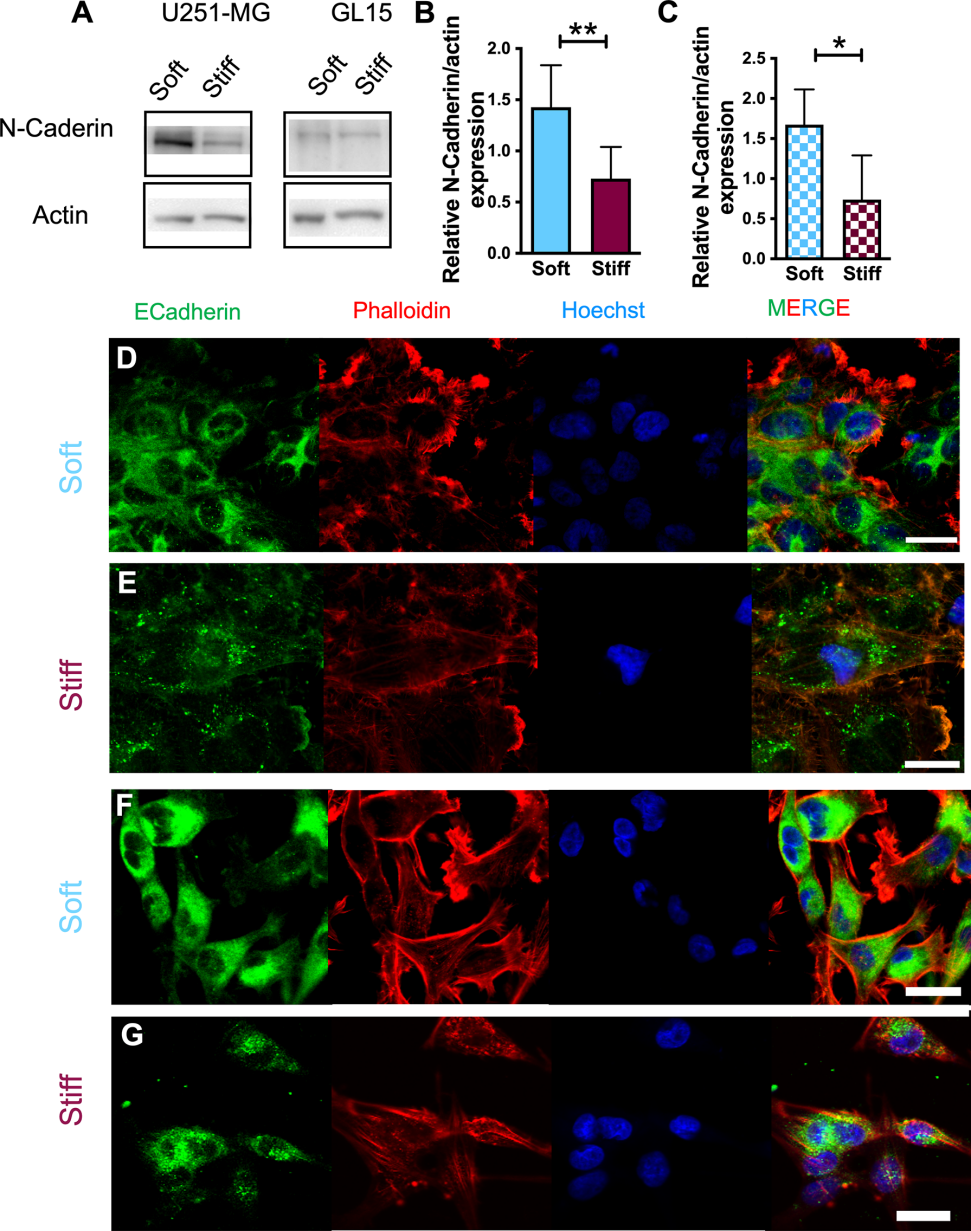
Expression of EMT hallmarks in U251-MG cells on substrates of different stiffness. **(A)** Representative western blot of four (n = 4) independent experiments and **(B, C)** relative quantification of N-Cadherin expression in U251-MG **(B)** and GL15 **(C)** cells on substrates of different rigidity. **(D-G)** Representative immunofluorescence images of U251-MG cells seeded on Soft **(D)** and Stiff **(E)** substrates and GL15 cells seeded on Soft **(F)** and Stiff **(G)** substrates. Cells were stained for phalloidin-TRITC (red), E-Cadherin (green) and Hoechst (blue). Scale bar 25 µm. Data presented as mean ± S.D. Student’s t-test was used for statistical analysis; *p < 0.05, **p<0.01.

Since we found a different response between cell migration sensitivity to substrate stiffness and phenotype, we explored the mechanism underlying this effect. E- and N-Cadherins have been previously identified as key players of the mechano-sensing pathway activated in response to different substrate stiffness or externally-applied forces (38). To specifically study the effect of stiffness on this cellular pathway, we analyzed expression of E- and N-cadherins in U251-MG and GL15 cell lines. Western Blot analysis revealed a downregulation of the E-Cadherin protein expression (data not shown) which is consistent with other studies which show that E-cadherin protein expression is commonly down regulated in GBM cells. Whereas N-cadherin expression was significantly increased on Soft substrates for both cell lines, as reported in **Figures 3A–C**, suggesting that less rigidity leads to the upregulation of N-cadherin.

As it has been proposed that E-cadherin molecules aggregate and anchor themselves to the actin filaments we further investigated its expression using confocal imaging. Fluorescence analysis using the E-cadherin monoclonal antibody, revealed a distinctly different expression between the two cell lines. In U251-MG, the E-cadherin signal showed a different localization in relation to the stiffness of the substrate. On Soft substrates a disorganized distribution on the plasma membrane, with localization around the nuclear region of cells and cell edges was observed (**Figure 3D**). Whereas, on Stiff substrates, no obvious labelling around the periphery was noticed but a more diffused pattern was seen (**Figure 3E**). In GL15, E-cadherin signal was localized on both cytoplasm and membrane regardless the rigidity of the substrates (**Figures 3F, G**) although on stiffer substrates localization was less homogeneous and more diffused. As the western blotting results of E-cadherin did not coincide with the immunofluorescence observations, we hypothesized this was caused to the co-presence of single cells and cell clusters and by loss of tight junctional staining at cell-cell contact areas supporting the down regulation in protein expression data and Western blot analysis, as previously reported (39).

As Cadherins actively interact with Catenins, which are membrane proteins that link the tail of Cadherins in the cytoplasm (i.e., N-Cadherin is associated with the actin cytoskeleton *via* β-Catenin expression), we also investigated β- Catenin expression on substrates of different stiffness through Western Blotting (**Supplementary Figure 1**). However, immunoblots of β-catenin levels, detected for both cell lines, did not significantly change with the rigidity of the substrate.

### EGFR expression is affected by substrate stiffness

**Figure 4:**
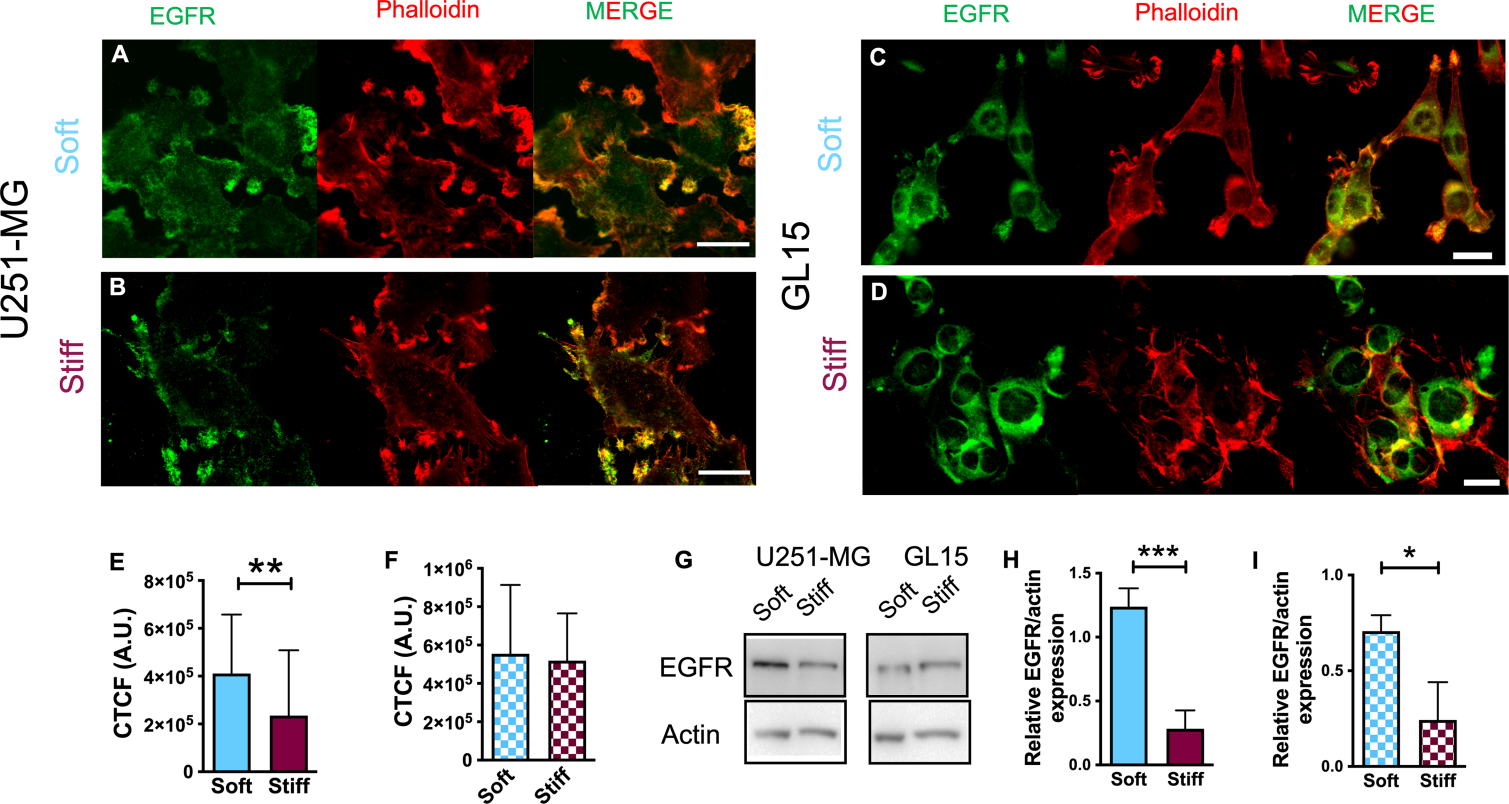
Decreased substrate stiffness stimulates EGFR expression in U251-MG. **(A–D)** Immunofluorescence images of U251-MG cells seeded on Soft **(A)** and Stiff **(B)** substrates and GL15 cells seeded on Soft **(C)** and Stiff **(D)** substrates. Cells were stained for phalloidin-TRITC (red), EGFR (green). Scale bar = 25 μm. **(E, F)** Quantification of fluorescence intensity of EGFR (expressed as CTCF, see methods section) of U251-MG **(E)** and GL15 **(F)** cells seeded on substrates of different stiffnesses. **(G)** Representative western blots of total EGFR levels in cells cultured on substrates of different stiffness. **(H**, **I)** Quantification of the immunoblots of U251-MG **(H)** and GL15 **(I)** cells on substrates of different stiffnesses. The percentage changes in the normalized EGFR levels were calculated relative to the normalized intensity for the petri condition (not shown). Measurements are representative of four distinct sets of data. Data, denoted as mean ± S.D., are representative of at least three independent experiments. Student’s t-test was used for statistical analysis; *p < 0.05; **p < 0.01; *** p< 0.001.

The activity of the EGFR pathway has been previously associated with changes in the microenvironmental stiffness (7, 21, 22). Therefore, we evaluated expression of EGFR in U251-MG and GL15 cells seeded on substrates with different rigidity. Confocal imaging confirmed distinctly different expression between the two cell lines. For U251-MG on Soft substrates, the detection of EGFR was largely diffused on the cell surface, but we also noticed an enrichment of the fluorescence signal at cell protrusions as shown in **Figure 4A**. Whereas on Stiff substrates, detection of EGFR expression was mainly observed on protrusions (**Figure 4B**). On the other hand, GL15 displayed an enrichment of the fluorescence signal at cell protrusions on softer substrates (**Figure 4C**) whereas on stiffer substrates it was more localized on the cell surface (**Figure 4D**). Quantitative analysis showed that the intensity of the fluorescence signal for U251-MG was lower on Stiff substrates compared to that of Soft (**Figure 4E**), without any significant difference for GL15 (**Figure 4F**).

Western blot analysis of EGFR protein expression (**Figures 4G–I**) confirmed an increased level of the EGFR on the Soft substrates respect to the Stiff substrates for both cell lines.

### Mechanical cues modulate ROS generation in GBM cells

**Figure 5:**
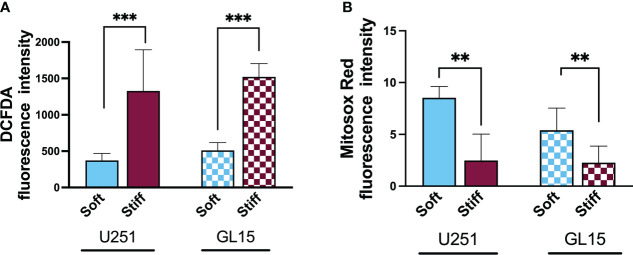
Substrate-influenced ROS generation in GBM cells. **(A)** Bar graphs showing the change of intracellular ROS production, detected with DCF-DA, in U251-MG and GL15 cells. **(B)** Bar graphs showing the change of mitochondrial ROS production in U251-MG and GL15 cells. Data presented as mean ± S.D.; n = 3 independent experiments for each condition. One-way Anova, applied for each cell line; *p<0.05, **p<0.01, ***p<0.001.

To evaluate whether the stiffness of the microenvironment could affect the alteration of cytoplasmic ROS in the GBM cells, we used the DCF-DA assay. This was carried out by measuring the oxidation of 2′,7′-dichlorofluorescein diacetate (DCF-DA) to fluorescent 2’,7’-dichlorofluorescein (DCF), as shown in **Supplementary Figure 2**. Analysis of the intensity of the fluorescence signal reported in **Figure 5A**, showed that the ROS baseline levels of both cell lines were significantly higher on the Stiff substrates respect to the Soft.

As ROS overproduction can trigger cell oxidative damage, to further quantify the contribution of the mitochondrial ROS to the total ROS amount in relation to the stiffness of the substrate, we used MitoSOX, a specific dye for mitochondrial ROS (**Supplementary Figure 3**). Quantification of the fluorescence signal, as shown in **Figure 5B**, showed a significant reduction in the mitochondrial ROS baseline level on stiffer substrates with both cell lines. These results point to the fact that the majority of endogenous ROS on the Soft substrates was generated in mitochondria.

Addition of the exogenous ROS source (H_2_O_2_) to culture medium showed an increased ROS production over time (24h) with an increased trend of DCFDA expression on Soft substrates for both cell lines (**Supplementary Figure 4**). Mitochondrial ROS with addition of H_2_O_2_ showed a trend towards increased intensity on soft substrates for U251-MG, whereas GL15 showed increased expression on stiff without significant differences (**Supplementary Figure 4**).

### Mitochondrial morphology and mitochondrial membrane potential are sensitive to substrate stiffness and cell types

**Figure 6:**
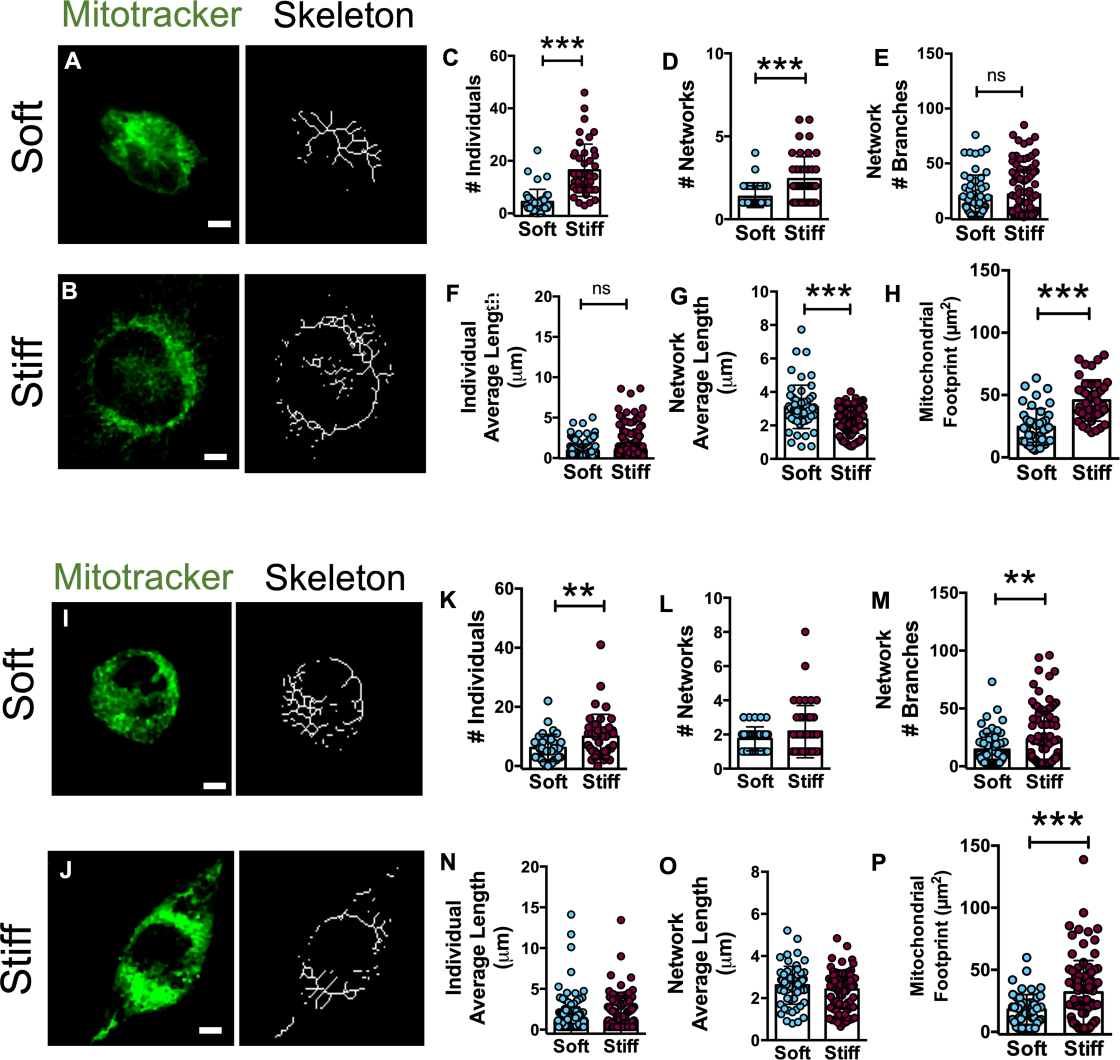
Mitochondria organization in U251-MG and GL15 cells is altered by substrate stiffness. Representative confocal images of U251-MG cells seeded on **(A)** Soft and **(B)** Stiff substrates and their relative skeletons. Scale bar 5 µm. Quantification of mitochondrial morphology showing the **(C)** number of individuals, **(D)** number of networks, **(E)** mean network size per branches, **(F)** mean length of branches/rod, **(G)** mean network size, and **(H)** mitochondrial footprint of U251-MG cells on Soft (n = 42 cells/8 fields) and Stiff (n = 43 cells/7 fields) and substrates. Representative confocal and skeleton images of GL15 cells seeded on **(I)** soft and **(J)** stiff substrates. Scale bar 5 µm. Quantification of mitochondrial morphology showing **(K)** the number of individuals, **(L)** number of networks, **(M)** mean network size per branches, **(N)** mean length of branches/rod, **(O)** mean network size, and **(P)** mitochondrial footprint of GL15 cells on Soft (n = 35 cells/6 fields) and Stiff (n = 42 cells/10 fields) substrates. The data are presented as mean ± S.D.; n = 3 independent experiments for each condition. Student’s t-test **p<0.01, ***p<0.001.

As mitochondria are the foremost active site for ROS production (40–42) and variations in mitochondrial shape are an indicator of mitochondrial activity, U251-MG and GL15 cells were coloaded with MitoTracker Green (43) and the mitochondrial structure was quantified using the Image J plugin Mitochondrial Network Analysis (MiNA) (31). This analysis allows to evaluate the number of unbranched puncta and rods structures (individuals) of the mitochondria and the branched structures (networks) and to identify the network complexity, expressed as the network branches, and the area occupied by the network, expressed as the mitochondrial footprint. Using this analysis, we compared the mitochondrial morphology of GBM cells on substrates of different stiffness (**Figures 6A, B, I, J**).

Quantitative analysis of the U251-MG skeletonized structures (**Figures 6C–H**) showed a decreased length of the network branches (**Figure 6G**) and a higher number of individuals (**Figure 6G**) and networks on the Stiff substrates (**Figure 6D**). In addition, the total area of mitochondria (footprint) was significantly increased on the Stiff substrates respect to Soft (**Figure 6H**). Similarly, GL15 exhibited a higher number of individuals (**Figure 6K**) and network branches (**Figure 6M**) on the Stiff substrates along with a higher area occupied by mitochondria of Stiff compared to Soft (**Figure 6P**). Collectively, these results suggest a more fragmented mitochondrial architecture in GBM cells on stiffer substrates.

**Figure 7:**
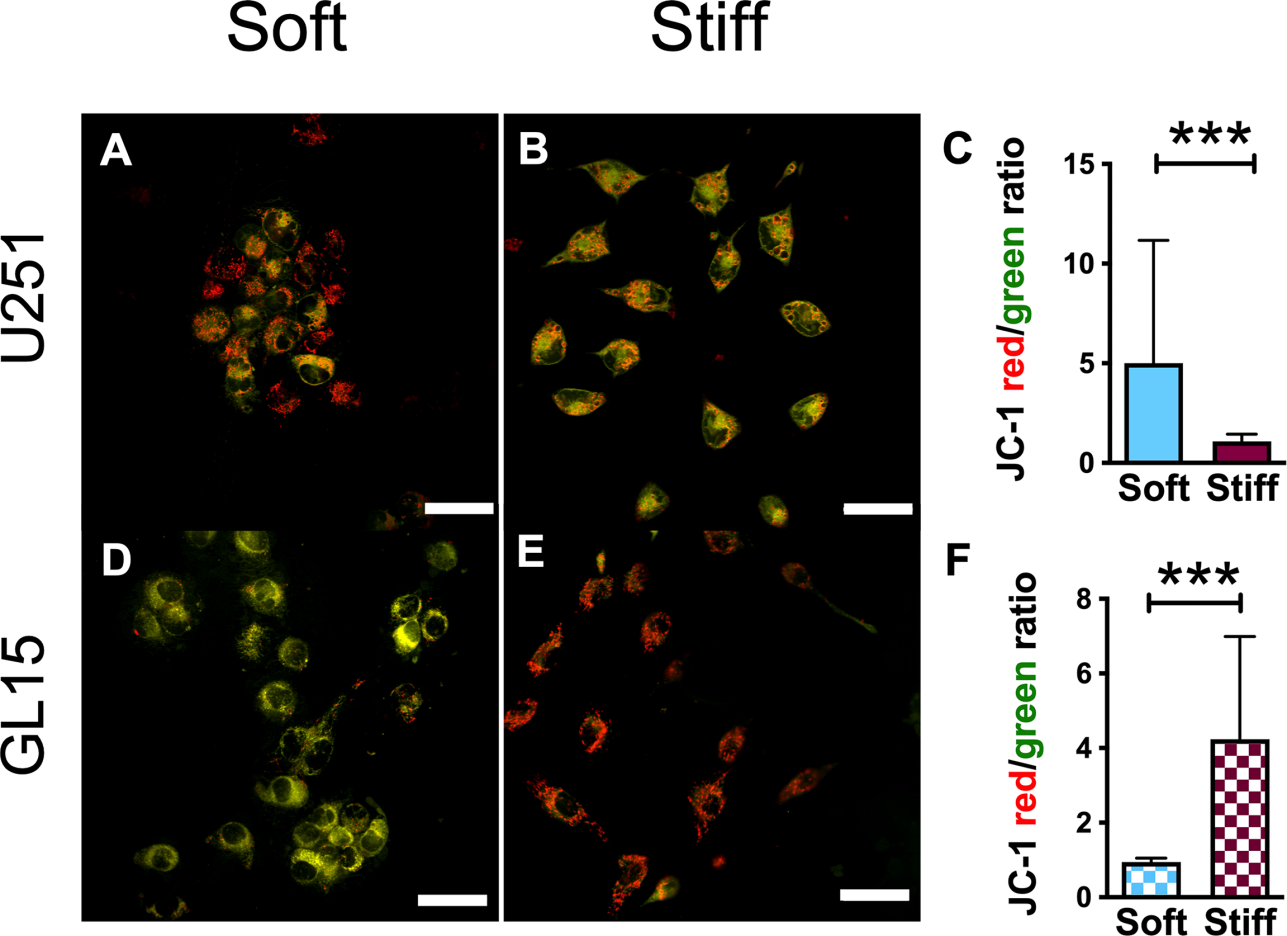
Mitochondrial membrane potential is rigidity- and cell type-dependent. **(A, B)** Representative confocal images showing JC-1 signals in U251-MG cells on Soft **(A)** and Stiff **(B)** substrates. The orange/yellow color denotes co-localization of red and green fluorescence signals. Scale bar 50 μm. **(C)** Quantitative analysis of ΔΨm, quantified as the ratio of red/green fluorescence intensity, in U251-MG cells seeded on the different substrates. **(D, E)**. Representative confocal images showing JC-1 signals in GL15 cells on Soft **(D)** and Stiff **(E)** substrates. Scale bar 50 μm. **(F)** Quantification of ΔΨm in GL15 cells seeded on the different substrates. Data presented as mean ± S.D.; n = 3 independent experiments for each condition. Student’s t-test; ***p<0.001.

To investigate how the changes in mitochondrial morphology can influence mitochondrial function, we assessed the effect of rigidity on the mitochondrial membrane potential (ΔΨm). ΔΨm is usually used as a marker of cellular health and mitochondrial membrane integrity, for instance decrease in ΔΨm is one of the premature events that leads to apoptosis (44). To this purpose, cells were stained with JC-1, a dye that exhibits potential-dependent accumulation in mitochondria. JC-1 usually aggregates (red fluorescence) in mitochondria of healthy cells, whereas monomers (green fluorescence) are produced in apoptotic cells. Results indicated a significant increase in the ratio of red/green fluorescence intensity for the U251-MG on the Soft substrates (**Figures 7A–C**), indicating that the Stiff substrates induced a depolarization of the mitochondrial membrane. On the contrary, we observed a higher red/green fluorescence intensity ratio on the Stiff substrates respect to Soft for the GL15 cells (**Figures 7D–F**). Our results show that matrix stiffness alone influences the ΔΨm in a cell dependent manner.

## Discussion

Different studies have suggested that GBM tumor tissues are stiffer than healthy brain, however, data reported is still contradictory, due to differences on the tumor source and measurement methods used. For instance, reports using confined compression (45) or ultrasound-based shear wave elastography (46) showed that GBM tumor tissues were stiffer than normal brain, whereas a study using shear compression reported no differences (47). Moreover, most reports agree that stiffness in the GBM tissue increases, but whether if it is ascribed to changes within the ECM, to an increased interstitial pressure, to the cells, or their combination is still under study (48). Also, numerous studies have reported the ECM stiffness to influence GBM invasion and proliferation, and to regulate cell cycle progression and proliferation through EGFR-dependent signaling and Phosphoinositide 3-Kinase (PI3K) expression (6, 7).

In this study, we investigated how the rigidity of the microenvironment influences the EMT and their interrelated pathways to determine a correlation with cell motility and the stiffness of the microenvironment. Since we observed a similar surface chemistry of substrates of different rigidity we can compare differences in pathway expression, between cell lines, excluding the chemical influence of the microenvironment. Moreover, since coating with proteins (i.e. collagen) has shown to penetrate deeper on softer substrates compared to stiffer (49), possibly affecting cellular responses, we chose to not use any coatings.

Cellular response to the microenvironmental stiffness may be very different in different cell types and depends on the nature of the adhesion receptor by which the cell binds its substrate such as cadherin-mediated intercellular junctions. For example, Pogoda reported that glioma cells (in particular LBC3 cells) on collagen-coated gels of stiffnesses of ~20 kPa showed the same area as on glass (50). However, on laminin-coated gels, cells exhibited a much smaller area, confirming a more chemically complex binding interface that involves not only the integrin targeted by the adhesive ligand used, but also the many serum and cell-derived factors that adhere to the substates used. Different glioma cell lines were reported on stiffer substrates to be non-spherical by extending F-actin rich lamellipodia and more mobile (6, 7, 51). We thus, explored the involvement of the molecular mechanism underlying the interplay among the cell response, cellular external microenvironment and EMT. The EMT is a process characterized by loss of cell-cell adhesion and the development of a spindle-like morphology (52). Our studies revealed that the two GBM cell lines on substrates of higher stiffness adopted a morphological mesenchymal phenotype. Examination of cytoskeletal rearrangements showed a clear remodeling of cell morphology related to the differences of the rigidity of their microenvironment, with a more spindled morphology on stiffer substrates and a marked round morphology, with generation of actin stress fibers, on Soft substrates for both cell lines. This is in correlation with previously reported studies which showed a change of the morphology of GBM cells through a mesenchymal phenotype of cells, with a higher stiffness of the substrate (6). Phenotypes more mesenchymal show higher invasiveness, whereas phenotypes more epithelial show a lower. We, however, did not detect a correlation between cell morphology and the increased migratory capacity of cells, as we observed a higher motility of U251-MG cells on stiffer substrates, whereas GL15 showed a decreased motility. Similar to our data, previous reports showed no correlation between the morphology and migratory invasiveness of cells of different GBM cell lines (51).

The difference in migration we observed could be due in part to the amount of protein present. Various studies, reported in literature, have shown that the ECM rigidity can modulate cytoskeletal configuration, protein expression, and signal transduction (53–56). Besides, the presence of an ECM protein has been shown to completely mask the effect of another, showing a potential crosstalk between proteins binding integrins (55, 56). We observed a higher protein absorption on stiffer substrates respect to Soft, in agreement with previous reports (36) but no significant differences were observed among coated and uncoated substrates. To avoid masking effects of the proteins we chose to use uncoated substrates. Moreover, as extensively renown, cells bind to different ECM proteins through different integrins (57). Different ECM proteins may bind to different integrins modulating the mechanosensitivity of cells and thus exhibit different responses to substrate stiffness. Thus, further studies varying the protein-coating concentration and integrin response will be investigated to examine this question.

Several studies also report controversial results with higher migration speed of GBM cells on stiffer substrates (6) whereas others reported decreased cell motility on stiffer substrate (58, 59). Actually, migrating tumor cells must mislay the mesenchymal phenotype to migrate to distant sites to form a secondary tumor (60, 61). Thus, EMT is a reversible process, and is most likely characterized by epigenetic alterations (51, 62) triggered by microenvironmental stimuli.

We, thus, further investigated the molecular involvement in the EMT process. EMT results from molecular changes involving phenotypical changes (loss of epithelial markers, as E-cadherin and increase of mesenchymal markers such as N-cadherin) (63–65). E-cadherin is rarely expressed in GBM. In fact, on the protein level, we did not detect E-Cadherin expression for both cell lines, consistent with most of the studies in literature reporting a lack of E-cadherin expression in normal brain, and in the majority of GBMs (13, 64, 66). Labelling E-cadherin in fluorescence showed a localization for U251-MG cells with a diffused presence over the entire surface of the plasma membrane on the Stiff substrates, whereas it was more concentrated in cytoplasmic areas, particularly in the peri-nuclear on Soft substrates. GL15 also showed a more concentrated incidence in cytoplasmic and peri-nuclear areas with a lower detection on stiffer substrates. The loss of endogenous E-cadherin, observed on stiffer substrates, can be associated to the triggering event of cell detachment from the primary tumor and its consequent invasive conduct (11).

Protein expression of N-cadherin was observed in both cell lines. In line with previous reports showing that cadherins are mechanosensors (67), we observed that N-cadherin expression was lower on substrates of increased stiffness. Studies showing a correlation between the invasive behavior and N-Cadherins are conflicting. Some reported that a decrease of N-cadherin was associated with a higher motility (11, 68), whereas others showed that its upregulation did not affect the degree of invasion (69). Our results confirm no association between expression of N-cadherin and GBM invasiveness, as in contrast with what occurs in epithelial tumors (13). More recent findings showed that a decrease of N-cadherin without re-expression of further cadherins is indicative of a loosening of cell–cell junctions denoting mechanical support for tumor cell migration away from the initial tumor (11).

As the expression of β-catenin contributes the progression of EMT we explored its expression, observing that the protein levels detected were independent from substrate stiffness (18). The mechanism employed by GBM cells to weaken cell–cell adhesion and enable migration and invasion may be related to alterations in the organization or processing of intercellular junction proteins rather than the regulation of their expression (70, 71). In fact, N-cadherin expression has been shown to influence position, number and size of focal adhesions (11), thus the turnover of focal adhesions on the different stiffness might be responsible for the different responses of cell speed that we observed in our results.

Several reports have also shown that E-Cadherin antagonizes EGFR activity, reporting that the downregulation of E-Cadherin triggers activation of EGFR, creating a positive reaction loop (22). Immunofluorescence performed with antibodies to EGFR showed that the localization of this receptor was dependent on the stiffness of the microenvironment. We observed a lower EGFR fluorescent localization and detection of protein level on Stiff substrates for both cell lines whereas levels of enrichment at the periphery of the cells, and throughout the cytoplasm were observed on Soft. This was in good agreement with previous reports showing E-cadherin enrichment at sites of cell-cell contact induced EGFR occurrence in sites of cell-cell contact (72). Moreover, a functional association between N-Cadherin and EGFR (7, 20, 22) can be confirmed.

Evidence also suggests a role of ROS in EMT and tumor aggressiveness (73, 74). Increased ROS levels can lead to the activation of different pathways that induce morphological changes related with the EMT. Assessing the effect of the microenvironment stiffness on cytosolic and mitochondrial ROS, we detected higher basal levels of cytosolic ROS and lower basal levels of mitochondrial ROS on stiffer substrates. Our data supports other recently published studies showing that substrate stiffness is a critical factor in modulating the intracellular ROS with an increase of cytosolic ROS levels of cells on increasing stiffness (29, 74, 75) as well as lower levels of mitochondrial ROS associated with induction of EMT.

As alterations in the redox status elicit mitochondrial fragmentation (42, 76), we analyzed mitochondrial morphology. Mitochondrial size and morphology, as well as their arrangement throughout the cells are organized through the fission and fusion of mitochondrial outer and inner membranes (77). Specifically, when mitochondria undergo fission, the network appears more fragmented, whereas, when enduring fusion, a more thin and elongated mitochondria is present. Besides, the mitochondrial morphology and function are linked to the cytoskeleton which in turn plays a major role in transducing mechanical signals. Little data is reported in literature related to whether ECM stiffness influences mitochondrial structure and function. We observed that the substrate stiffness significantly influences the numbers of mitochondrial individuals and networks. Specifically, both cell lines (U251-MG and GL15) showed larger mitochondria footprint on the Stiff substrates and number of individuals. This is in line with recent evidence reporting a more fragmented morphology of cells on stiffer substrates (75). In this respect, we hypothesize that the increase in mitochondrial network length of U251-MG on the Soft substrates may be representative of a cell-protective compensatory mechanism, active under metabolic stress conditions.

Mitochondrial fragmentation has been postulated to coordinate with mitophagy to repair deficiencies of both structural and functional integrity of the mitochondrion with reduced mitochondrial membrane potential or increased ROS production (78). Our findings point to a negative correlation between mitochondrial membrane potential and cell migration. In fact, U251-MG presented a depolarized ΔΨm together with an increased motility on the stiffer substrates, whereas ΔΨm of GL15 cells was lower on Soft, where they are more most motile, suggesting that the change of extracellular mechanical environment modulates motility and consequently energy production, metabolism and signaling.

Though the crucial role of the mechanical microenvironment has been widely established, studies till now, have analyzed the pathways, reported here separately. Our data unifies response of several pathways and signals that induce EMT, to the rigidity of the microenvironment and suggest that increasing the stiffness of the cell niche, promotes EMT. Increasing the ECM stiffness promotes a behavior typical of an EMT-induced condition and a cadherin switch, which may be indicative of the transition from a benign to an invasive, malignant tumor phenotype. In effect, a stiffer microenvironment supports cells detachment and migration to distant sites. However, we observed that the migratory abilities are independent of EMT. Moreover, our data point to a regulation of GBM cells based on microenvironmental cues transmitted by integrin and extracellular matrix proteins, as well as how the signals eventually translate to metabolic modifications coupled with changes in cell migration. EGFR is renowned to act with integrins in regulating cell–ECM interactions, and some integrins act as redox sensors. Moreover, previous reports showed that the shape and function of the mitochondria is influenced by interactions with a variety of cytoskeletal proteins in an integrin-dependent manner (79). Thus, further studies should point to uncovering ECM remodeling and integrin arrangement of primary cells as well an in a 3 D microenvironment.

Understanding the molecular basis underlying EMT in a more physiological study, may aid to the identification of new molecular targets for rationally designed therapies. Future studies examining the influence of Ca^2+^ signaling events, as well as the crosstalk with growth factor signaling and the Wnt pathways will be important and, hopefully, will shed new light on how the mechanical cues influence the invasiveness of GBM cells. We believe that our current findings may aid to identify novel signaling pathways that link to the mechano-sensing influence, opening to development of new therapeutic interventions targeting tumor invasiveness.

## Data availability statement

The raw data supporting the conclusions of this article will be made available by the authors, without undue reservation.

## Author contributions

BB, IP and BC conceived and designed the study. BC, PR, FC, CS and SD’A carried out experimental work. CL, MR, MG completed the western blot processing and analysis. BB carried out the mitochondrial analysis. MC completed AFM analysis. GG, DR, SDA supervised and edited the manuscript. All authors revised the manuscript and read and approved the submitted version.

## Funding

The research leading to these results has received funding from AIRC under IG 2021 - ID. 26328 project – P.I. Cortese Barbara and AIRC under MFAG 2015 - ID. 16803 project – “P.I. Cortese Barbara”. The authors are also grateful to the ”Tecnopolo per la medicina di precisione” (TecnoMed Puglia) - Regione Puglia: DGR n.2117 del 21/11/2018, CUP: B84I18000540002 and “Tecnopolo di Nanotecnologia e Fotonica per la medicina di precisione” (TECNOMED) - FISR/MIUR-CNR: delibera CIPE n.3449 del 7-08-2017, CUP: B83B17000010001.

## Acknowledgments

We thank Dr. Francesca Pagani for useful technical support. We thank also Irene Iacuitto, Giovanna Loffredo and Manuela Marchetti for practical administrative support.

## Conflict of interest

The authors declare that the research was conducted in the absence of any commercial or financial relationships that could be construed as a potential conflict of interest.

## Publisher’s note

All claims expressed in this article are solely those of the authors and do not necessarily represent those of their affiliated organizations, or those of the publisher, the editors and the reviewers. Any product that may be evaluated in this article, or claim that may be made by its manufacturer, is not guaranteed or endorsed by the publisher.
