# Supplementary material for: Substrate stiffness effect on molecular crosstalk of epithelial-mesenchymal transition mediators of human glioblastoma cells
Source: Front Oncol. 2022 Aug 25;12:983507. doi: 10.3389/fonc.2022.983507 (PMC9454310; doi:10.3389/fonc.2022.983507)
Supplement: Supplementary file 1 [file DataSheet_1.docx]

Supplementary Material

Substrate stiffness effect on molecular crosstalk of Epithelial-Mesenchymal Transition mediators of Human Glioblastoma Cells

**
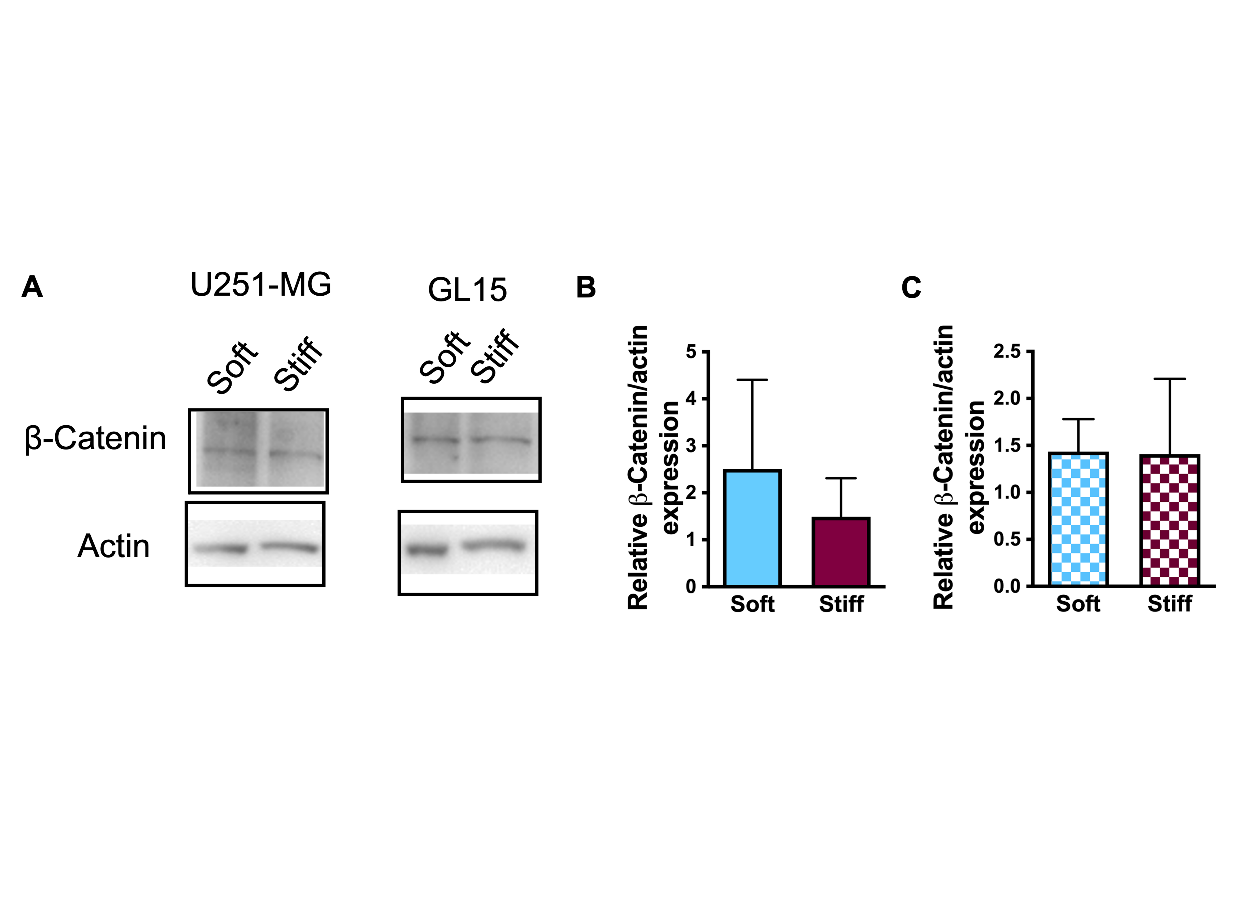
**

**Supplementary Figure 1.** (A) Representative western blot and (B-C) relative quantification of β-Catenin expression in U251-MG (B) and GL15 (C) cells on substrates of different rigidity. Data presented as mean ± S.D., n=3 independent experiments for each condition. Student’s t test was used for statistical analysis.


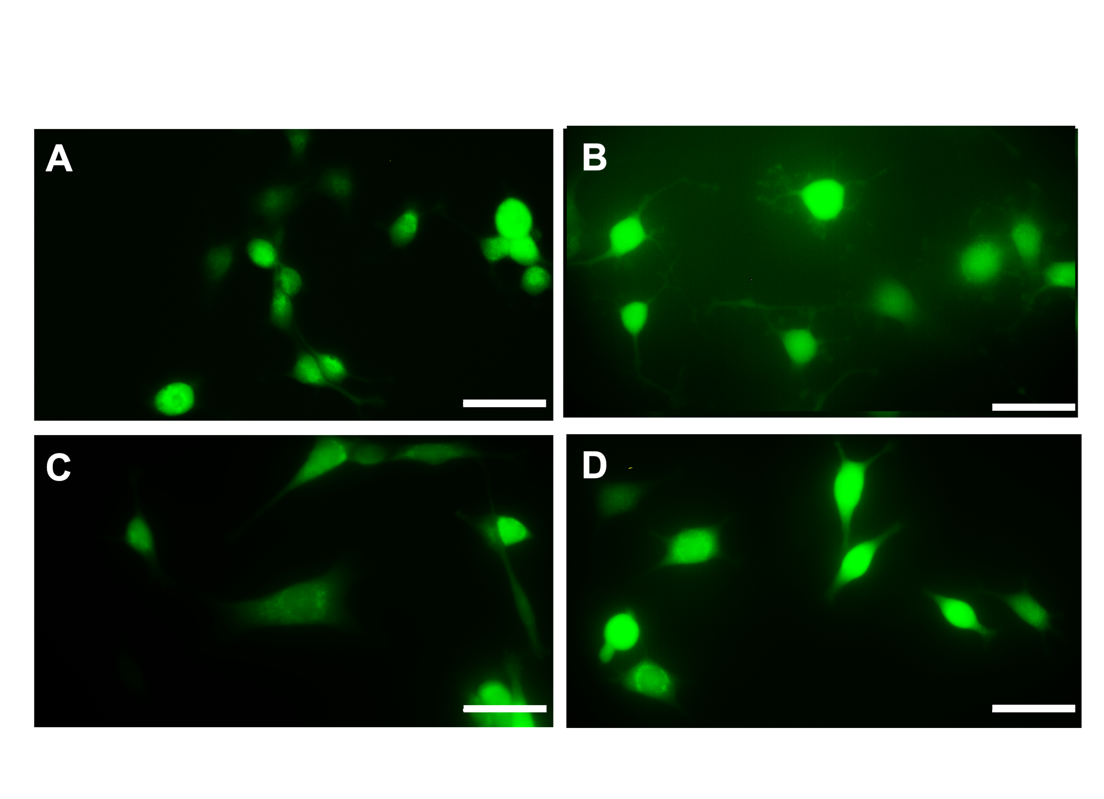

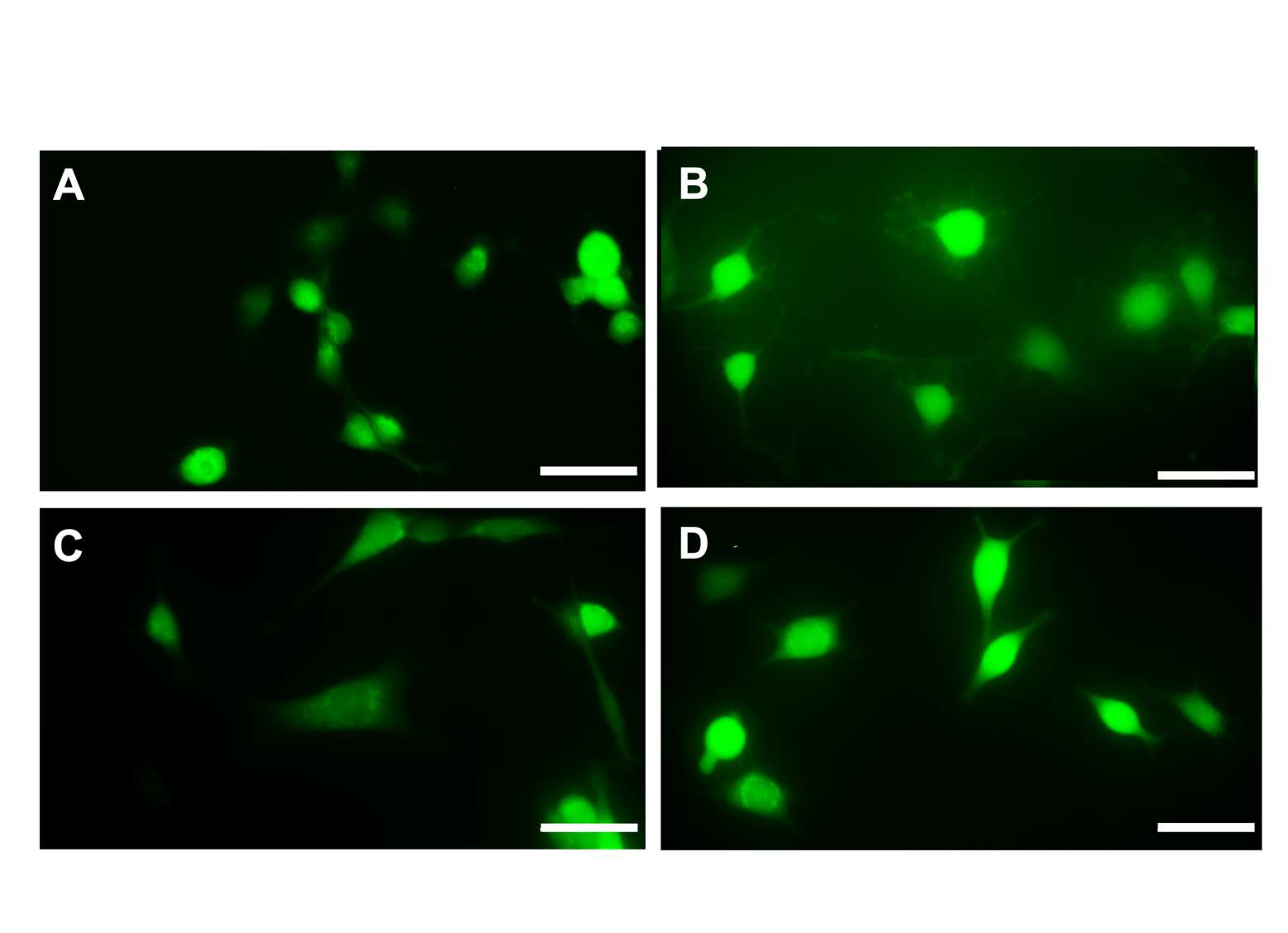


**Supplementary Figure 2.** Substrate-induced different ROS generation in glioblastoma cells. Representative confocal images stained with DCF-DA of U251-MG on Soft (A) and Stiff (B) substrates, and GL15 on soft (C) and stiff (D) substrates. Scale bar 50 µm.


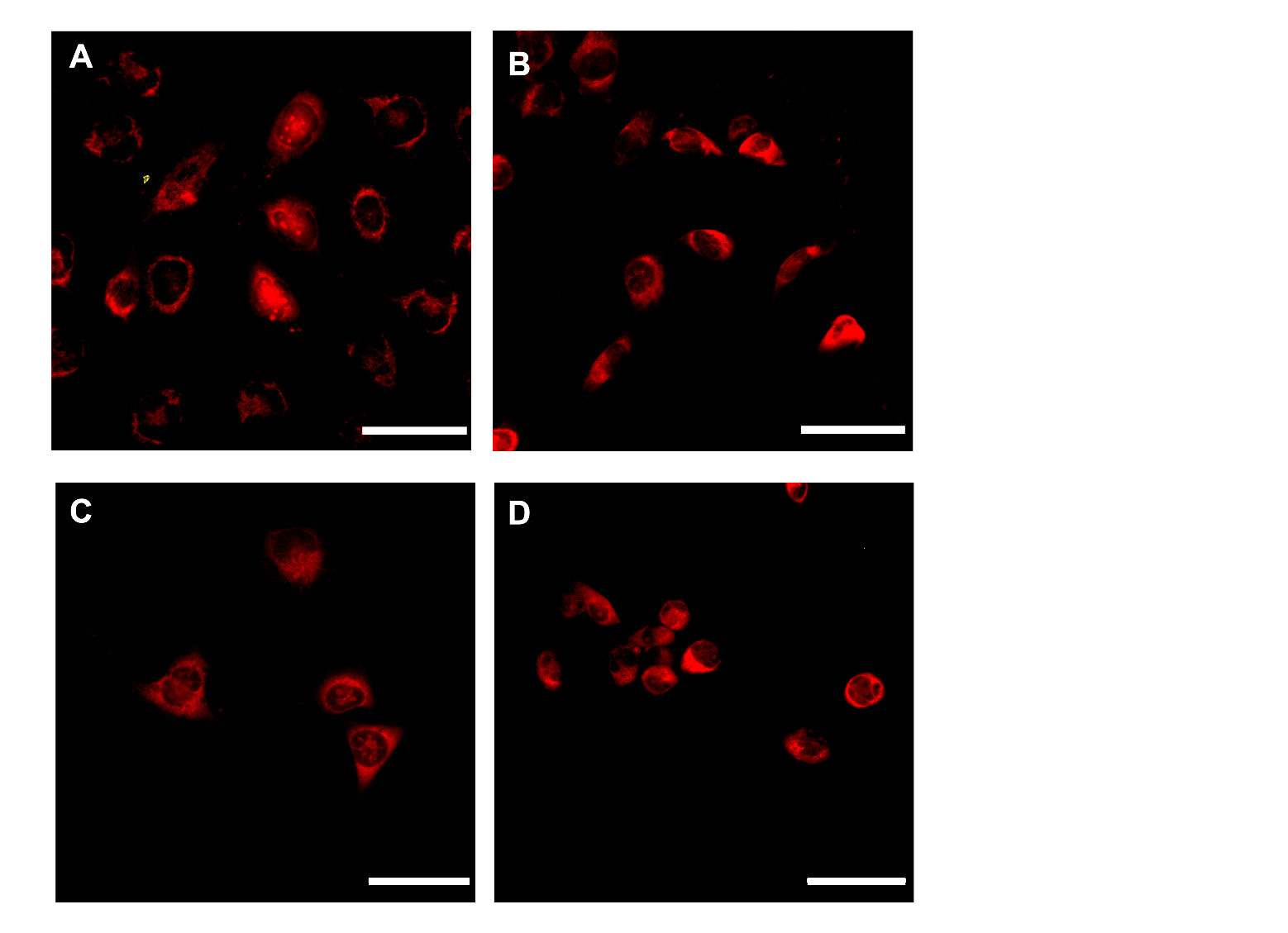


**Supplementary Figure 3.** Substrate-induced different Mitosox ROS generation in glioblastoma cells. Representative confocal images stained with MitoSOX Red of U251-MG on Soft (A) and Stiff (B) substrates, and GL15 on Soft (C) and Stiff (D) substrates. Scale bar 50 µm.


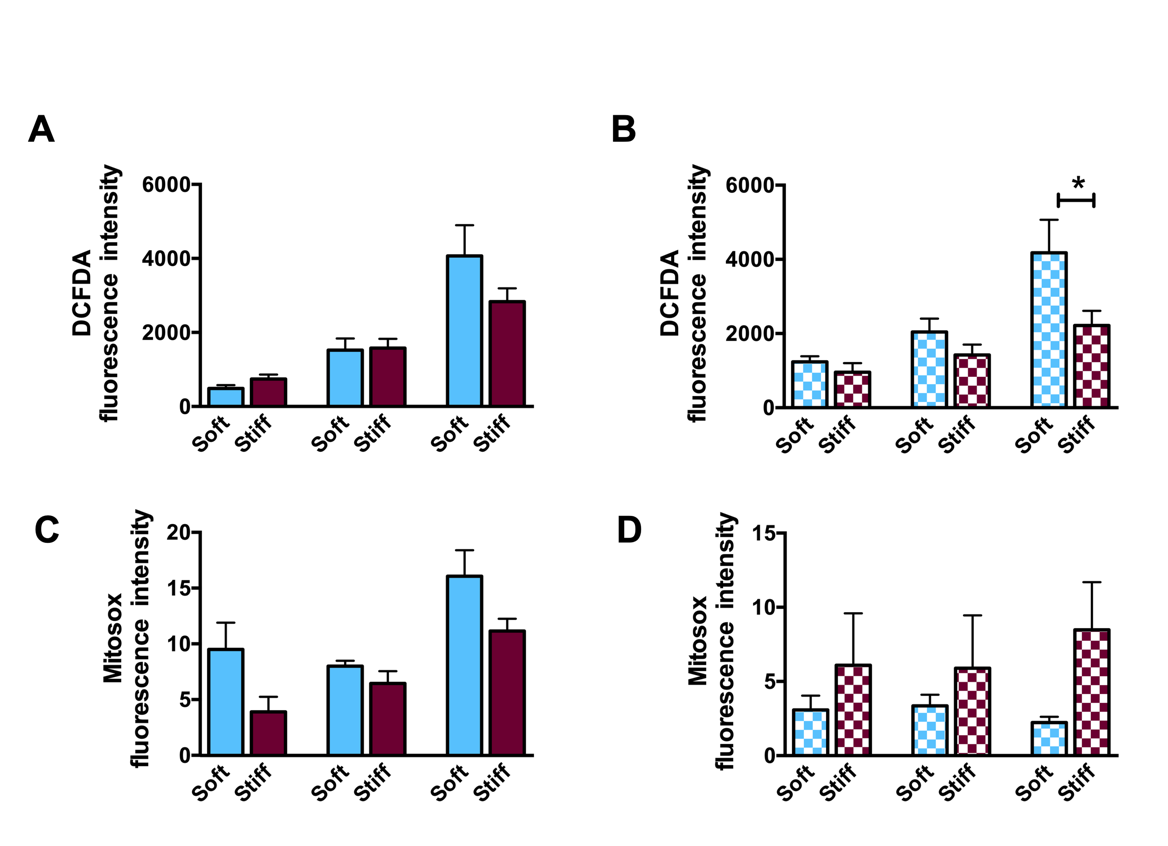


**Supplementary Figure 4.** Bar graph showing H_2_O_2_-induced ROS levels measured by (A) DC-FDA and (B) MitoSOX Red staining in U251-MG and (C) DCF-DA and (D) MitoSOX Red staining in GL15. Data presented as mean± S.E.M. Data were compared using a Two-way ANOVA (n=3 independent experiments) significant differences *p<0.05.
